# Factors predicting a successful post-discharge outcome for individuals aged 80 years and over

**Published:** 2012-02-10

**Authors:** Line Kildal Bragstad, Marit Kirkevold, Dag Hofoss, Christina Foss

**Affiliations:** Faculty of Medicine, Department of Nursing Science, Institute of Health and Society, University of Oslo, P.O. Box 1130 Blindern, NO-0318 Oslo, Norway; Faculty of Medicine, Department of Nursing Science, Institute of Health and Society, University of Oslo, P.O. Box 1130 Blindern, NO-0318 Oslo, Norway; Faculty of Medicine, Department of Nursing Science, Institute of Health and Society, University of Oslo, P.O. Box 1130 Blindern, NO-0318 Oslo, Norway; Faculty of Medicine, Department of Nursing Science, Institute of Health and Society, University of Oslo, P.O. Box 1130 Blindern, NO-0318 Oslo, Norway

**Keywords:** post-discharge outcomes, family caregivers, care transitions, aged 80 and over

## Abstract

**Introduction and background:**

The early post-discharge period is a vulnerable time for older patients with complex care requirements. This paper identifies factors predicting a self-reported successful post-discharge outcome for patients aged 80 years and over by exploring factors related to the discharge process, the provision of formal home-care services, informal care and characteristics of the patients.

**Methods:**

The study reports results from survey interviews with patients admitted from home to 14 hospitals in Norway and later discharged home. Logistic regression analysis was performed to assess the impact of a number of factors on the likelihood that the patients would report that they managed well after discharge.

**Results:**

The odds of managing well after discharge were more than four times higher (OR=4.75, p=0.022) for patients reporting that someone was present at homecoming than for those who came home to an empty house. Patients who reported receiving adequate help from the municipality had an odds four times (OR=4.18, p=0.006) higher of reporting that everything went well after discharge than those who stated the help was inadequate.

**Conclusions:**

Having someone at home upon return from hospital and having adequate formal home-care services are significantly associated with patient-reported success in managing well.

## Introduction

Older patients with multiple and often complex care requirements are being discharged from hospital to home ‘quicker and sicker’ than ever before, and thus at an earlier stage of the rehabilitation process [1–3]. The early post-discharge period is an especially vulnerable phase which involves significant transitions for older patients and their family caregivers [4–6]. Furthermore, today’s health-care systems have an objective to ensure that older persons are able to live at home as long as possible [7] and to reduce the need for admission to care institutions.

During the last 20 years we have seen a substantial change in policy resulting in a general downscaling of care institutions in Norway and other European countries [8, 9]. To compensate for this deinstitutionalization there has been an expansion of the municipal home-care services in Norway [7] and a steady increase in the overall number of formal home health-care recipients [10]. However, taking into account the population growth over the same period, there was a proportional decrease from 41% of the 80 and over age group receiving home-care services in 1992 to 37% in 2006 [7, 10]. Furthermore, patients aged 80 and over are on average granted fewer service hours than patients aged 67 and under [11]. These contemporary changes in the primary and secondary health-care services call for further exploration. This paper identifies factors that may predict a self-reported successful post-discharge outcome for patients aged 80 and over.

## Theory

**Figure 1:**
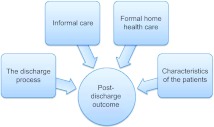
Four groups of factors assumed to influence post-discharge outcome in the transition process from hospital to home for patients aged 80 years and over.

Several literature reviews have identified factors influencing the transition process and post-discharge outcomes [5, 12–15]. Professional/service factors, informal/family caregiver factors, personal factors [5] and factors related to discharge planning [13] were found to be crucial to the transition process between hospital and home. As shown in Figure 1, these four groups of factors are assumed to influence the post-discharge outcome.

### The discharge process

Hospital professionals are commonly in charge of discharge planning; however, participation by professionals from the primary health-care services jointly with family caregivers is required to make transitions from hospital to home as efficient and safe as possible [9]. The goal of discharge planning is to prepare patients and their family caregivers for life at home following hospitalization [15]. In order to feel prepared to return to their homes, patients express a need for information and arrangements regarding care issues, activities of daily living and where to turn if unforeseen events arise [16]. During the early post-discharge period, defined as the first three to five weeks, approximately 20% of the oldest patients experience adverse events [17, 18]. This may be indicative of unsuccessful discharge and could potentially lead to re-admission to hospital or transfer to a nursing home. Studies have shown that a relatively short length of hospital stay [19] and living at home rather than in sheltered accommodation [19, 20] increases the probability of readmission. Discharge planning combined with additional post-discharge support can reduce unplanned readmission [13].

### Characteristics of the patients

Essential personal factors include readiness for discharge [5, 16], level of disability and subsequent need for post-discharge support [5]. Difficulties with activities of daily living tend to increase with advancing age. Old age is associated with a high prevalence of mulitimorbidity, chronic illness, as well as sensory and functional impairment and a general decline in health [2, 18, 21–24]. Physiological changes associated with ageing predispose older patients to serious complications at the time of hospital discharge and following it [24]. Frailty of patients or significant deterioration in functional status, as well as the presence of cognitive problems, can be predictive of unsuccessful post-discharge outcomes [3, 25, 26]. Most patients experience increased functional dependency post-discharge and hence require formal post-hospital home-care [27], often in conjunction with extensive informal care from unpaid carers [28].

### Formal home-care services

Coming home from hospital, older patients need emotional support and require assistance with personal and instrumental activities of daily living [2]. In Norway and other Nordic countries the welfare state holds the main responsibility for the care of older people [29, 30]. The municipal home-care services in Norway provide both formal home-help services and round the clock home-nursing care. Allocation of home-care services in Norway is not limited to a set time period, but is based on individual needs assessments. Service hours are allocated depending on the patient’s needs, and can be adjusted when necessary. On average, patients aged 80 and over were allocated 4.65 hours per week in 2010 [11]. Home-care assistants in the home-help services usually provide assistance with personal care activities, such as bathing, dressing, feeding and instrumental activities of daily living. Administering medication, giving injections and changing wound dressings, on the other hand, are examples of tasks carried out by home nurses. Formal home-care delivery in Norway is viewed as generous compared to other countries [31]. However, studies from countries with comparable health-care systems—Canada [2] and the UK [32]—have shown that home-care services may be inadequate in meeting the full range of the patient’s post-discharge needs.

### Informal care

Family members, neighbours and friends are essential informal care providers when older patients return home after hospitalization [6, 12, 28, 29, 32–35]. Patients receiving extensive formal care from the municipalities in Norway continue to receive informal care from family caregivers [29, 36, 37]. Estimates show that close to 80% of the home care in Norway [35] and the UK [32] is provided by family members and other informal caregivers. Formal and informal caregivers complement each other and provide help with different tasks [29]. Formal caregivers have been found to perform personal activities of daily living, while family caregivers or other informal caregivers offer help with instrumental activities of daily living [29]. Family caregivers have always had a leading role in helping older people at home [6]. However, in Norway the welfare system is built on the premise that public health care should be sufficient, and older people should not have to rely on informal caregivers to manage. The deliberate shift away from hospital care towards home-care has intensified the pressures on families and increased their role in supporting older people after discharge [15, 32].

### Research question

A clear emphasis on the importance of recognising patients as experts with a unique knowledge of their own health and preferences has emerged through the policy initiatives and health-care legislation of recent years [38, 39]. Surveys to ascertain patients’ views serve as tools to elicit information that contributes to improved practices [40]. Research also supports the notion that seeking patients’ views and preferences in the discharge process is of vital importance for a successful discharge [41]. The specific research question we seek to answer in this study is therefore:

How do the patient-reported discharge process, formal home-care, informal care and state of health influence the patients’ self-reported post-discharge outcome?

## Methods

### Background and sample

**Figure 2:**
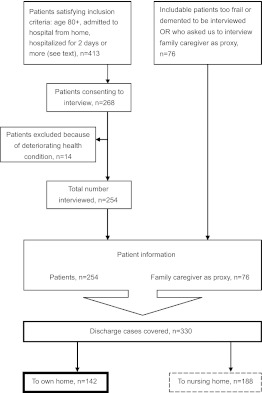
Flow chart of inclusion of respondents and discharge cases covered in the study.

The study is part of a research project funded by the Norwegian Research Council, in which self-reported questionnaire results for patients admitted from home to 14 hospitals in Norway and discharged home to long-term community care are reported. The charge nurses at home-care offices in 67 Norwegian municipalities identified potential participants and introduced the study to patients who met the inclusion criteria. Inclusion criteria were: aged 80+, admitted to hospital from home, hospitalized for 2 days or more and adequate cognitive performance (as assessed by the recruiting nurse) to take part in the planning of their own discharge and to give written informed consent to participate in the study. Three hundred and thirty respondents were recruited to the main study (Figure 2).

At the time of the interview, 43% (142) of the 330 respondents in this study lived at home while 57% (188) were nursing-home residents. The sample in this paper consists of the 142 home-dwelling patients.

### The questionnaire

The Discharge of Elderly Questionnaire was developed by the research team. It was designed to elicit data about the patients’ experiences regarding their discharge and the management of their health problems after discharge. There was no existing questionnaire covering these dimensions [42]. The questionnaire was organized in four main parts: ‘Here-And-Now’, ‘At the Hospital’, ‘Summary’ and ‘Demographic Background’. The ‘Here-And-Now’ section contains questions about how the patient manages after discharge. ‘At the Hospital’ is divided into six subcategories: ‘Information about the hospital stay’, ‘the discharge process’, ‘received information and training’, ‘participation in the discharge planning’, ‘communication’ and ‘the role of family caregivers’. In the ‘summary’ part of the questionnaire patients were asked concluding questions about their general assessment of the help received during their hospital stay. The last section of the questionnaire concerns the patients’ demographic background, previous and current care arrangements and present functional status. Functional status was measured by four ADL-measures (dressing, bathing, transferring and feeding) [43] and three IADL-measures (shopping, light household chores and heavier household chores) [44]. Performance was graded as independent, partly dependent or dependent.

### Data collection

Geriatric nurses or geriatric nurse students carried out structured face-to-face interviews with the patients during the first two weeks following discharge from hospital. Family caregivers interviewed as proxy were interviewed by telephone. Interviewers were trained to clarify the questions in a uniform way, and to help respondents grade their answers [45].

### Data analysis

**Figure 3:**
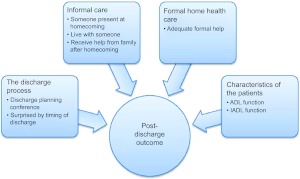
Variables in our data material organized in four groups of factors assumed to influence post-discharge outcome in the transition process from hospital to home for patients aged 80 years and over.

Logistic regression analysis was performed to assess the impact of a number of factors on the likelihood that the patients would report that they managed well after being discharged home from hospital. The independent variables ‘adequate help from the municipal home health care’, ‘someone was present when I came home’, ‘I live alone’, ‘I receive help from family now’, ‘there was a discharge planning conference’, ‘I was surprised by the timing of my discharge from hospital’, ADL sum and IADL sum were included in the logistic regression model (Figure 3).

The analysis was controlled for age, gender and length of hospital stay. The p-value of the Hosmer and Lemeshow model for goodness of fit was p=0.894. An α-level of 0.05 was used in all statistical tests. Data were analysed using PASW Statistics 18.

### Ethical considerations

The study was designed in accordance with the World Medical Association’s Declaration of Helsinki [46]. Approval for the study was obtained from East Norway Regional Ethics Committee for Medical Research (project number: 17078) and all the municipalities involved. Informed written consent was obtained from each patient before the interviews were initiated.

## Results

### Characteristics of the sample

In our sample of 142 home-dwelling patients with a mean age of 85.9 years, 70.4% (100) were women (Table 1).

Thirteen (9.4%) of the patients had been in education beyond upper secondary school. While 29.1% (41) of the patients were married, 62.4% (88) were widows or widowers. At the time of the interview 66.2% (92) of the patients lived alone.

### Managing after discharge

As shown in Table 2, 54.1% (66) of the patients reported that they had managed well after their homecoming. This response is interpreted as a self-reported successful post-discharge outcome.

In 91.2% (93) of the cases, no discharge planning conference was held. Furthermore, 20% (24) reported that the timing of their discharge from hospital surprised them. Statements made by the patients (Table 3) suggest that some were surprised because they thought they were discharged too early and they wanted to stay in hospital until they felt strong enough to return home.

A family member was present at the patient’s homecoming in 57.7% (71) of the cases. In 12.2% (15) of the cases someone from the home-care services was present, yet 15.4% (19) of the patients came home to an empty house (Table 4). Thirteen (10.6%) of the patients reported that they did not require any assistance at homecoming. Patient statements (Table 3) show that some of the patients were prepared for coming home to an empty house, and did not experience this as a problem. However, some patients felt lonely and abandoned, and others shared experiences of difficulties managing on their own.

At the time of the interview 80.3% (114) of the patients reported that they received help from their family. In our sample 93.7% (133) of the patients received home-nursing care. In addition, 67.6% (96) of the patients received home-help. Despite this, 28.4% (35) of the patients reported that they felt the help they received from the municipality was not adequate. Patient statements (Table 3) suggest that the feeling of inadequacy stems from what they feel is an insufficient allocation of service hours and a need for more help with IADL tasks like grocery shopping and house cleaning.

As shown in Table 5, two of the independent variables made a unique statistically significant contribution to the logistic regression model.

Controlled for the other factors in the model, the odds of managing well after discharge were more than four times higher (OR=4.75, p=0.022) for patients reporting that someone was present when they came home than for those who came home to an empty house. Patients reporting that they thought the help they received from the municipality was adequate had an odds four times (OR=4.18, p=0.006) higher of reporting that everything went well after discharge than those who thought the help was inadequate. The patients’ age, gender, length of stay, ADL and IADL function, whether they received help from family and friends, lived alone, reported being surprised by the timing of the discharge or whether they reported that there was a discharge planning conference were not statistically significant predictors in this model.

## Discussion

In our study, having someone at home upon returning from hospital was an important predictor for a self-reported successful post-discharge outcome. The patients were met at their home by family members in 57.7% of the cases and by others in 16.3% of the cases. The family’s involvement commences early in the transition process, preparing and assisting in the homecoming for the patients. Our findings suggest that it is imperative for a successful post-discharge outcome that the patient does not come home to an empty house.

Another important predictor for a self-reported successful post-discharge outcome was having adequate formal home health care. In our sample all patients received formal home-help and/or home-nursing care. However, 28.4% of the patients found the formal help insufficient. Earlier research has pointed towards the inadequacy of municipal home-care services [2, 32]. In our study we are unable to pinpoint precisely what the patients found insufficient. But statements made by the patients suggest that the need for social support in addition to practical help with instrumental activities of daily living is perhaps the one need not commonly met by formal caregivers in today’s ‘stopwatch service’ provision. To promote a feeling of well-being and mastery after coming home, it seems to be important for the municipality to perform an assessment of the patients’ needs for services that correspond to the patients’ own expectations.

As earlier research has shown, informal help from family and friends is an important supplement to the formal home help provided by the municipality [6, 12, 28, 32–35]. In our sample 80.3% of the patients received help from family and friends. Our findings, supported by patients stating ‘it would not have gone this well without my daughter’ and ‘the home nurses and my wife are helping me’ (Table 3), highlights the importance of both the informal and formal caregivers at homecoming.

In our logistic regression model ADL and IADL function were not statistically significant with regard to the dependent variable. That is not to say that the patient’s functional status does not affect the post-discharge outcome, it probably just means that the patient’s functional dependency was compensated for by the amount of formal and informal help received post-discharge.

Despite the fact that 91.2% of the patients reported that there was no discharge planning conference and that 20% reported being surprised by the timing of their discharge, the logistic regression model did not confirm our assumption that these variables are significant predictors of a successful post-discharge outcome. However, these findings raise questions that need further exploration concerning the quality of the discharge planning and the cooperation between formal and informal caregivers regarding the patient’s discharge.

The capacity in the Norwegian home-care sector is under pressure [9] and the findings from this study indicate that both informal care and formal home health care are vital elements for older patients discharged from hospital.

## Conclusion

Our findings show that having someone at home upon returning from hospital and having adequate formal home-care services are significantly associated with patient-reported success in managing well in the long-term after returning home from hospital.

## Figures and Tables

**Table 1.** Characteristics of the sample

Discharged to own home, 43% (n=142/330)	
Length of hospital stay	
Mean	10.4 days
Median	7 days
Time since discharge	
Mean	16.7 days
Median	14 days
ADL-sum^1^ (S.D.)	
Mean	10.5 (1.79)
Median	11
IADL-sum^2^ (S.D.)	
Mean	4.9 (1.83)
Median	5
Age	
Mean	85.9 years
Median	85 years
	% (n)
Gender	
Women	70.4 (100)
Men	29.6 (42)
Marital status	
Married	29.1 (41)
Widow/widower	62.4 (88)
Divorced	3.5 (5)
Cohabiting	0.7 (1)
Unmarried	4.3 (6)
Level of education	
Primary school	46 (64)
Lower secondary/vocational school	38.8 (54)
Upper secondary school	5.8 (8)
University or college degree	9.4 (13)
Living status	
Alone	66.2 (92)
With someone	33.8 (47)
Type of residence	
Private, not adapted	42.8 (59)
Private, adapted	26.8 (37)
Municipal housing, adapted	29 (40)
Other	1.4 (2)

^1^ADL-sum ranges from 4—dependent in all activities to 12—independent in ail activities. ^2^IADL-sum ranges from 3—dependent in all activities to 9—independent in all activities.

**Table 2.** Self-reported post-discharge outcome

How have you managed since coming home from hospital?	% (n)
It has been okay all along	54.1 (66)
It was difficult at first, but okay after a while	18.9 (23)
It has been mixed (difficult and okay) all along	16.4 (20)
It has been difficult all along, and I still find it difficult	9.8 (12)
My experience does not fit in any of the categories	0.8 (1)
Total^1^	100 (122)

^1^Total number of patients discharged to own home were 142. For various reasons family caregivers were interviewed as proxy for 19 of the patients. Proxies were not asked to answer this question, thus, the total number of respondents who were asked this question was 123. One person did not answer the question, resulting in a total number of 122 answers.

**Table 3.** Examples of patient statements

***Question***	***Typical statements—patient quotes***	
How have you managed at home since your discharge?	Well	“I have received a lot of help, my son is visiting” “It has been okay all along thanks to the home nurses” “The home nurses and my wife are helping me” “It would not have gone this well without my daughter”
	Not well	“I have not been well, very dizzy and powerless” “I feel tired and weak, and the home nurses are not here long enough” “I think I was discharged too early considering my health status” “I have had some pain, it has been difficult to walk” “I feel lonely after coming home”
If you came home to an empty house, how was that experience for you?	Good	“It was okay, I didn't need someone there” “It was okay, I had my telephone and TV. I have always lived alone, so I'm used to it” “I knew I would be on my own at home, it was okay”
	Bad	“No one was there. No one was there to say, “welcome home”. The mailbox was full. But the home care aide came and helped me to bed” “I was too tired to “feel anything”, I fell asleep in my chair. The taxi driver helped me to my living room” “I felt lonely and abandoned. I had a dream that the home care aide would be there ready with a cup of coffee” “It was very difficult. I had great pain in my hip, and I had to walk the stairs to my house. Luckily, a neighbor came to my assistance” “On account of a misunderstanding the hospital's discharge notice failed to reach my family. That's why I came to an empty house. I was able to reach my family, and they came shortly after.”
If the formal help you receive is insufficient, what would you want differently?		“I would like to exercise more” “I could use some more physical therapy” “It is not enough and the job they do is often unsatisfactory” “I need more help with laundry and window cleaning. I am lonely” “I wish someone could do my grocery shopping” “I need help with house cleaning” “I only get help with one shower per week” “I wish I could get more than two hours per week now that I am ill”
Did the timing of the discharge surprise you?	No	“I was prepared” “I was told the same day, but felt prepared” “No, I was prepared they wouldn't let me stay long, despite me feeling weak and weary”
	Yes	“I felt I was too ill to go home” “I thought they would run more tests and that the stay would be longer. I was very ill” “I wanted to stay at the hospital longer” “I had not been told what was wrong with me, I was surprised. They took our beds in the morning, and I had to sit on a chair waiting for the taxi until 5 pm. It was horrible” “Yes, and because of that I asked to stay longer, but my request was declined”

**Table 4.** Homecoming

Was someone present when you came home from the hospital?	% (n)
Not necessary, I can manage on my own	10.6 (13)
No, I came home to an empty house	15.4 (19)
Yes, my next of kin was present	57.7 (71)
Yes, someone from the formal home health services was present	12.2 (15)
Someone else was present	4.1 (5)
Total^1^	100 (123)

^1^Total number of patients discharged to own home was 142. For various reasons family caregivers were interviewed as proxy for 19 of the patients. Proxies were not asked to answer this question, thus, the total number of respondents who were asked this question was 123.

**Table 5.** Logistic regression model

	B (S.E.)	p-Value	Odds ratio (95% CI)
Gender (0=female)	0.396 (0.514)	0.411	1.486 (0.543–4.070)
Age	–0.090 (0.056)	0.110	0.914 (0.819–1.021)
Length of stay	–0.026 (0.025)	0.298	0.974 (0.927–1.024)
ADL-sum^1^	–0.246 (0.166)	0.140	0.782 (0.565–1.084)
IADL-sum^2^	0.076 (0.149)	0.608	1.079 (0.806–1.446)
Adequate help from municipality (0=no)	1.430 (0.518)	0.006	4.177 (1.514–11.526)
Someone present when I came home (0=no)	1.558 (0.682)	0.022	4.749 (1.248–18.078)
Live alone (0=yes)	0.525 (0.520)	0.313	1.690 (0.610–4.682)
Help from family now (0=no help)	–0.885 (0.600)	0.140	0.413 (0.127–1.337)
Discharge planning conference (0=no)	0.513 (0.995)	0.606	1.671 (0.238–11.752)
Surprised by discharge (0=yes)	0.903 (0.576)	0.117	2.467 (0.797–7.634)
Constant	7.736 (5.350)	0.148	2288.178

*The dependent variable: self-reported post-discharge outcome (0=the first 2–3 weeks after discharge from hospital were difficult in the beginning, but ok after a while/both difficult and ok all along/difficult all along and still difficult, 1=ok all along). ^1^ADL-sum ranges from 4—dependent in all activities to 12—independent in all activities. ^2^IADL-sum ranges from 3—dependent in all activities to 9—independent in all activities. (Hosmer and Lemeshow model goodness of fit p=0.894) (n=122).

## Reviewers

**Omar Hasan**, MBBS, MPH, MS, Medical Director, Continuum of Care Strategies, Brigham and Women's Hospital, Boston, MA, USA

**Carol Levine**, MA, Director, Families and Health Care Project, United Hospital Fund, 1411 Broadway, 12th Floor, New York, NY 10018-3496, USA

**Ingalill Rahm Hallberg**, Professor in Health Care Science; president of the European Academy of Nursing Science (EANS); Assistant Vice Chancellor, Lund University, Sweden

**Olle Söderhamn**, RN PhD, Professor (Nursing Science) University of Agder, Faculty of Health and Sport Sciences, P.O. Box 509, NO-4898 Grimstad (Norway); and Centre for Caring Research—Southern Norway.
